# Supplementary material for: Optimization Studies and Compositional Oil Analysis of Pequi (Caryocar brasiliense Cambess) Almonds by Supercritical CO2 Extraction
Source: Molecules. 2023 Jan 19;28(3):1030. doi: 10.3390/molecules28031030 (PMC9920824; doi:10.3390/molecules28031030)
Supplement: Supplementary file 1 [file molecules-28-01030-s001.zip › molecules-2094817-supplementary.pdf]

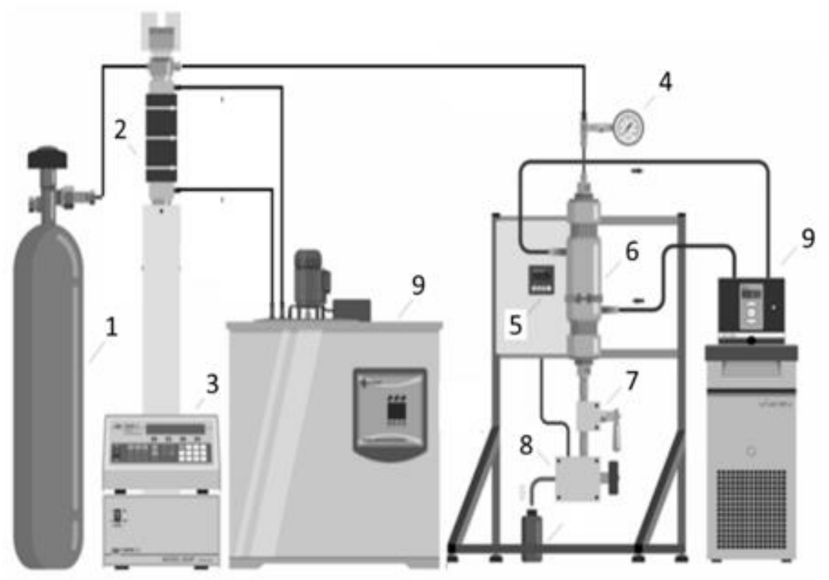

Figure S1. Schematic of the experimental supercritical extraction unit:1-CO_2 cylinder; 2-Syringe pump; 3-Thermostatic bath; 4-Pressure indicator; 5-Temperature controller/indicator; 6-Extractor; 7-Valve; 8-Needle-type valve attached to an aluminum jacket for heating; 9-Thermostatic bath.
